# Supplementary material for: MetaQTL: a package of new computational methods for the meta-analysis of QTL mapping experiments
Source: BMC Bioinformatics. 2007 Feb 8;8:49. doi: 10.1186/1471-2105-8-49 (PMC1808479; doi:10.1186/1471-2105-8-49)
Supplement: Additional File 7 — MetaQTL Package : jar file and tutorial. This Zip archive contains both the MetaQTL JAR file and the files of the tutorial. [file 1471-2105-8-49-S7.zip › org.inra.metaqtl/doc/org/metaqtl/bio/IBioEntity.html]

IBioEntity


|  |  |  |
| --- | --- | --- |
| **Overview** \| **Package** \| **Class** \| **Use** \| **Tree** \| **Deprecated** \| **Index** \| **Help** | |  |
| **PREV CLASS**   **NEXT CLASS** | **FRAMES**    **NO FRAMES**     **All Classes** |  |
| SUMMARY: NESTED \| FIELD \| CONSTR \| METHOD | DETAIL: FIELD \| CONSTR \| METHOD |  |


---


## org.metaqtl.bio Interface IBioEntity

**All Superinterfaces:**: IBioAdaptable

**All Known Subinterfaces:**: IBioAllele, IBioCross, IBioGenome, IBioIndividual, IBioLGroup, IBioLocus, IBioOntology, IBioOntologyTerm, IBioPopulation

**All Known Implementing Classes:**: AlleleContainer, BioEntity, BioEntityCollection, BioEntityContainer, CrossPopulation, GeneticMap, GroupContainer, Individual, IndividualContainer, LGroup, Locus, LocusContainer, Marker, Ontology, OntologyTerm, Population, QTL

---

``` public interface IBioEntity extends IBioAdaptable ```

Class Description Here

**Author:**
:   Jean-Baptiste Veyrieras

---

| **Method Summary** | |
| --- | --- |
| `java.lang.String` | `getName()`             Returns the name of the biological entity |
| `IBioEntity` | `getParent()`             Returns the entity that handles this entity. |
| `java.util.Properties` | `getProperties()`             Gets the properties of the entity. |
| `int` | `getType()`             There are 2 main class of entities. |
| `void` | `setName(java.lang.String value)` |
| `void` | `setProperties(java.util.Properties defaultProps)`             Sets the properties of the entity. |

**Methods inherited from interface org.metaqtl.bio.IBioAdaptable**

`getBioAdapter`

**Method Detail**

### getName

```
java.lang.String getName()
```

:   Returns the name of the biological entity

---


### setName

```
void setName(java.lang.String value)
```

---


### getType

```
int getType()
```

:   There are 2 main class of entities. The first one deals with population
    biological entity, i.e population itself and individuals. The second class
    is a representation of microscopic biological entity from genome container
    to alleles.

---


### getParent

```
IBioEntity getParent()
```

:   Returns the entity that handles this entity.

---


### getProperties

```
java.util.Properties getProperties()
```

:   Gets the properties of the entity.

---


### setProperties

```
void setProperties(java.util.Properties defaultProps)
```

:   Sets the properties of the entity.

    :   **Parameters:**: `defaultProps` -


---


|  |  |  |
| --- | --- | --- |
| **Overview** \| **Package** \| **Class** \| **Use** \| **Tree** \| **Deprecated** \| **Index** \| **Help** | |  |
| **PREV CLASS**   **NEXT CLASS** | **FRAMES**    **NO FRAMES**     **All Classes** |  |
| SUMMARY: NESTED \| FIELD \| CONSTR \| METHOD | DETAIL: FIELD \| CONSTR \| METHOD |  |


---
